# Scapulothoracic Bursitis in a Patient With Quadriparesis: A Case Report

**DOI:** 10.1097/MD.0000000000000752

**Published:** 2015-04-24

**Authors:** Seung Jun Seol, Seung Hoon Han

**Affiliations:** From the Department of Rehabilitation Medicine, Hanyang University College of Medicine, Seoul, Korea.

## Abstract

Scapulothoracic bursitis is a rare disease and presents as pain or swelling around the bursa of the scapulothoracic articulation. It has been reported to be related to chronic repetitive mechanical stress of the periscapular tissue, trauma, overuse, and focal muscle weakness. The authors experienced an atypical case of scapulothoracic bursitis with shoulder and periscapular pain after quadriparesis.

This case implies that muscular atrophy around the scapula and chest wall from quadriparesis may contribute to the development of scapulothoracic bursitis with shoulder and periscapular pain. In addition, clinician should be alert to it as a possible cause when a patient with quadriparesis complains of shoulder and periscapular pain and consider proper diagnostic options such as ultrasonography or magnetic resonance imaging.

## INTRODUCTION

A scapulothoracic bursitis is a rare disease; its pathogenesis is known to be related to chronic repetitive mechanical stress of periscapular tissue, usually resulting from a bone abnormality such as a protrusion of the scapula or rib cage.^1^ Conduah et al^2^ reported that scapulothoracic bursitis could be caused from an inflammation of the bursa secondary to trauma or overuse owing to sports activities or work. They also reported that focal muscle weakness or atrophy could also play a role in idiopathic cases of scapulothoracic bursitis.^2^ Fujikawa et al^1^ reported a case of chronic scapulothoracic bursitis after thoracoplasty, and El-Khoury et al^3^ reported 2 cases of bursa formation in association with osteochondromas.

Individuals with spinal cord injury may experience muscle atrophy because of their reduced metabolic rate and disuse of affected body.^4^ Periscapular muscle atrophy is seen in chronic patients with quadriplegia and it may increase friction force between thoracic wall and scapula. Increasing mechanical stress may also contribute to the development of scapulothoracic bursitis or aggravation of the preexisting bursitis. In the authors’ knowledge, this is the first report of scapulothoracic bursitis in a patient with quadriparesis and the authors report the case with related literature review.

## METHOD

This is a case report and approval of the ethics committee or institutional review board was not obtained. Informed consent was not obtained from the patient because he was not trackable after discharge.

## CASE DESCRIPTION

A 59-year-old man was referred to the authors’ department with complaints of aggravated left shoulder and periscapular pain. He suffered from left shoulder and periscapular pain for several years. He could not exactly remember when his pain started; however, he guessed that it had been >5 years. He took pain medications to control the pain during 5 years; however, intensity of pain did not change. He had been diagnosed with quadriparesis caused by an incomplete cervical myelopathy after falling down 30 years ago. He had alert mental status with good cognitive function. When he was referred to the department of rehabilitation, he could walk with moderate assistance, although he appeared frail and had very thin body. A month prior, he was hospitalized in the Department of Thoracic Surgery, Hanyang University College of Medicine, Seoul, Korea, due to left fourth to ninth rib fracture and hemothorax caused by a bicycle accident. A chest tube was inserted to resolve the hemothorax and conservative therapy was continued.

After his general chest condition improved, he was transferred to the Department of Rehabilitation to evaluate aggravated left shoulder and periscapular pain since admission. He had vague pain in the left posterior shoulder and inner surface of the scapula. However, he could not precisely explain or point out the exact area that was painful. He could not sleep well due to increased pain at night, and his score on the visual analog pain scale was 7 points.

On physical examination, generalized atrophy was observed in the left shoulder girdle muscles, and there was remarkable atrophy of the infraspinatus muscle. On inspection, the inner area of the left scapular medial border appeared slightly edematous; however, there was no erythema or tenderness. Muscle strength of all extremities was grade 4 on the Medical Research Council scale; however, left shoulder abductor, flexor, extensor, and intrinsic muscles of both hands were weakened to grade 3. He also showed spasticity of 1+ grade of the modified Ashworth scale on all extremities. Hyperactive deep tendon reflexes were evoked on bilateral elbow, knee, and ankle joints. Upper motor neuron signs, such as Hoffman and Babinski signs, were also present on bilateral extremities. He also had paresthesia such as tingling sensation below bilateral C5 dermatome. No deformities, scars, or specific tender points were noted on the left shoulder.

Because he had several clinical findings of cervical myelopathy, the authors recommended the patient to undergo cervical spine magnetic resonance imaging (MRI) and electrodiagnostic test to clarify his disease status; however, the patient did not want to work up for it. Although he had no radiologic and electrodiagnostic data to prove cervical myelopathy, the authors were able to diagnose him with chronic myelopathy based on his past history and supporting clinical findings.

**FIGURE 1:**
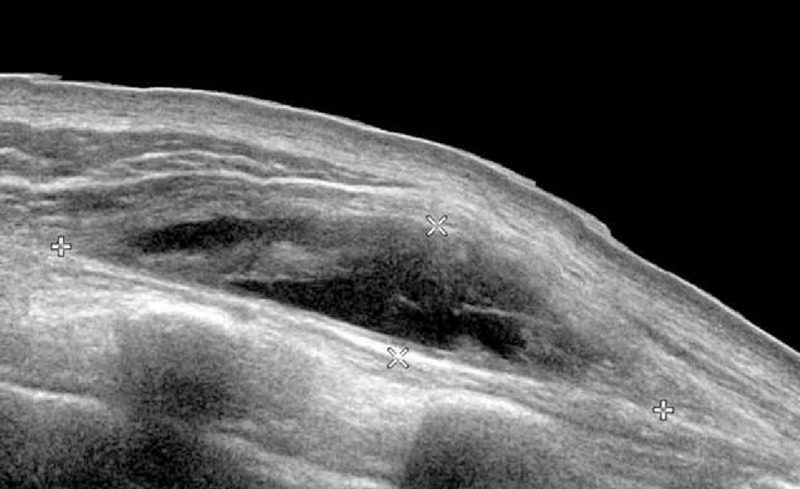
Longitudinal view of left shoulder on ultrasonography and periscapular area shows heterogenous fluid-filled cystic mass.

**FIGURE 2:**
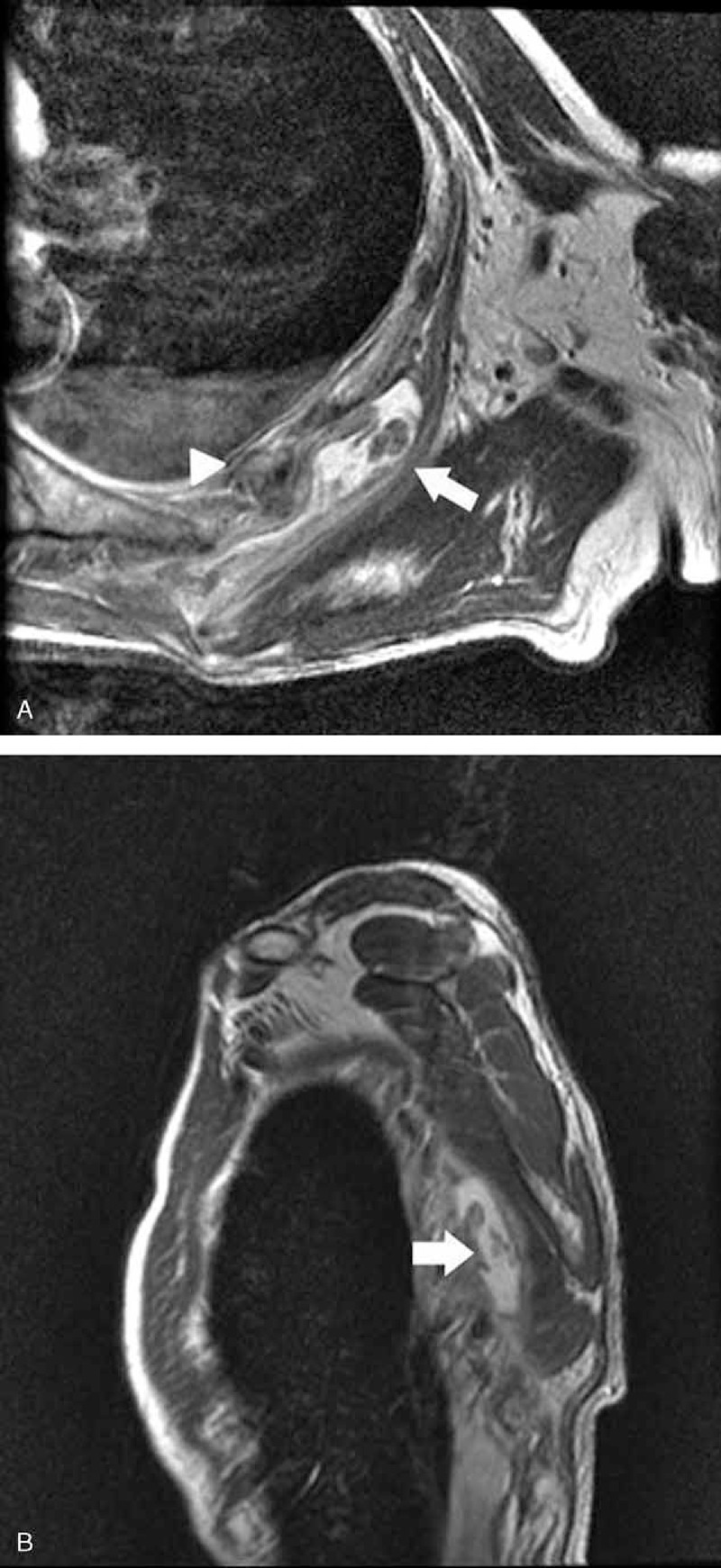
High signal intensity in enlarged scapulothoracic bursa (arrow) and fractured rib (arrowhead) in (A) axial and (B) sagittal view of T2-weighted magnetic resonance images.

First, the authors reviewed simple chest and left shoulder radiographs, and no special conditions, other than multiple left rib fractures, were found. Musculoskeletal ultrasonography was then performed to rule out subacromio–subdeltoid bursitis or a rotator cuff tendon tear. However, ultrasonography revealed a scapulothoracic bursa fluid collection with debris between the serratus anterior muscle and chest wall (Fig. 1) measuring approximately 6.6 × 3.6 × 1.5 cm. Chest MRI was recommended for detailed evaluation (Fig. 2). On chest MRI, there was no evidence of tendon or muscle injury, and a 10 × 7 × 6 cm heterogeneous fluid-filled cystic mass lesion was found between the serratus anterior muscle and the rib cage at the level of inferior pole of the left scapula. The wall of the mass was thin and uniform, with papillary projections; however, there was no communication between fractured ribs and the cystic mass. Based on these findings, the mass was diagnosed as chronic scapulothoracic bursitis rather than hematoma secondary to trauma.

Conservative treatment including oral medication and physical therapy was immediately started. Acetaminophen and tramadol were used to control pain, and physical modalities including hot pack, ultrasound, and transcutaneous electrical nerve stimulation were applied on the left shoulder. Range of motion and strengthening exercise of left shoulder girdle muscles were also performed. These treatments continued for 4 weeks. Needle aspiration and injection could not be done due to the debris in the bursa. The pain gradually decreased and the score of visual analog pain scale was 2 points. In addition, it did not worsen after stopping analgesic medication. The muscle strength of the left shoulder abductor, flexor, extensor, and intrinsic muscles of both hands had not been changed during the treatment. Edema around the left scapular wing greatly improved after 4 weeks of treatment. It was recommended to the patient that he undergo follow-up imaging study such as ultrasound or MRI to view possible improvement; however, the patient declined imaging due to the high financial burden of the proposed studies and was discharged.

## DISCUSSION

Scapulothoracic bursitis presents as pain or swelling of the bursa at the scapulothoracic articulation.^5^ It is thought to be related to chronic repetitive mechanical stress caused by bony abnormalities of the scapula or rib cage.^1^ Muscular imbalance^5^ or osteochondroma^6^ around the scapula are causes of such mechanical stress, and a sudden distended scapulothoracic bursa could be related to hemorrhage.^7^ In this case, although the patient could walk with moderate assistance, he had muscular atrophy around the scapula and chest wall secondary to chronic cervical myelopathy and had multiple rib fractures that may have contributed to the distension of the bursa.

Before shoulder ultrasonography, the authors hypothesized that the rib fracture caused by trauma could be related to the swelling on the inner left scapular surface. Physical examination, such as inspection of fullness of the bursa around the scapula, palpation of the bursa with attention to tenderness, and scapulothoracic crepitus, should be performed during evaluation of a patient with suspected scapulothoracic bursitis.^5^ In this case, physical examination findings were limited to edema near the left scapular medial border without tenderness. Unfortunately, the examining physicians did not evaluate the patient for pain and crepitus with scapulothoracic motion because scapulothoracic bursitis was extremely rare in the clinical setting.

Ultrasonography was very helpful in revealing the type and location of scapulothoracic bursa at an early stage. In this case, ultrasonography initially showed a large scapulothoracic bursa located between the serratus anterior muscle and the rib cage near the inferior angle of the scapula. Although MRI is the best tool for revealing the exact anatomy of soft tissue pathology, its use is limited due to its high cost. Thus, ultrasonography may be a good imaging alternative in patients with suspected scapulothoracic bursitis, and can be performed with high accuracy by experienced or trained physicians.

Two major and 4 minor bursae of the scapulothoracic articulation have been described previously. Infraserratus and supraserratus bursae accounted for the 2 major bursae; the 4 minor bursae were 2 bursae on the superomedial angle of the scapula, a bursa on the inferior angle of the scapula, and a trapezoid bursa on the spine of the scapula.^5^ In the case of the patient presented here, scapulothoracic bursa was located around inferior angle of the scapula and distended to the upper area. Muscular atrophy around the scapula and chest wall might increase friction between the serratus anterior muscle and the rib cage, resulting in a large scapulothoracic bursa. In addition, bony abnormalities of the chest cage after rib fracture might have aggravated the status of preexisting bursitis.

Higuchi et al^7^ described 9 cases of distended scapulothoracic bursitis. All 9 patients presented with a painless palpable mass below the scapula, and the initial diagnoses were soft tissue tumors. Hemorrhage was present within the bursa in all cases and every mass regressed in size spontaneously during a period of a few to several weeks. Ken et al^8^ also reported 4 cases of scapulothoracic bursitis. None of the patients described in this study had pain, tenderness, warmth, or erythema. The diameters ranged from 6 × 6 to 20 × 15 cm. Two were treated with only conservative treatment, and 1 patient underwent a successful surgical removal. The fourth patient was treated with aspiration. The masses disappeared in all cases by the time of the final follow-up. Fujikawa et al^1^ reported a case of surgical resection of chronic scapulothoracic bursitis caused after thoracoplasty in a woman with left inferior periscapular pain. At the time of surgery, a partial rib resection was performed because of a firm, fibrous adhesion between the mass and the adjacent ribs. The characteristics of the previous reported cases are summarized in Table 1. In this case, the bursa size was measured as 10 × 7 × 6 cm and it contained heterogeneous fluid that was thought to be hemorrhage. The authors assumed that hemorrhage might be induced from rupture of papillary projections inside the bursa wall based on MRI findings of no direct communication between the bursa and the fractured ribs. Abrupt hemorrhage inside the wall may enlarge the size of the bursa and aggravate shoulder and periscapular pain.

**TABLE 1:**
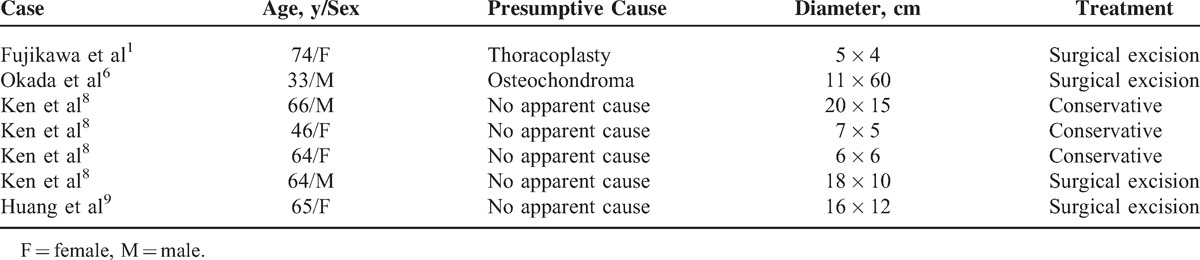
Characteristics of Previous Reported Cases of Scapulothoracic Bursitis

Generally, conservative treatment includes rest, local modalities such as hot packs, ultrasound, and transcutaneous electrical nerve stimulation, shoulder exercise, anti-inflammatory drugs, and intracystic injection of long-acting corticosteroids or ethanol.^9^ If an osseous lesion accompanies the mass or the patient fails conservative treatment, surgical intervention can be considered.^9^ Fortunately, conservative treatment, including medication and physical therapy, was very effective at relieving pain in this case, and the patient refused additional treatment and was discharged.

The authors think that there are some limitations of this report. First, the authors did not suspect scapulothoracic bursitis as a cause of his shoulder pain at the time of patient's admission. Therefore, the authors did not perform physical examinations related to scapulothoracic bursitis such as evaluating pain and crepitus with scapulothoracic motion. Second, the authors could not evaluate the long-term effect of treatment because the patient was satisfied with the result of treatment and did not come to the authors’ department after his discharge.

Nevertheless, this case is worth introducing because it is the first report that indicates muscular atrophy after quadriparesis as a cause of scapulothoracic bursitis and subsequent shoulder pain. In addition, this case implies that the clinicians should be alert to scapulothoracic bursitis as a cause if a patient with quadriparesis complains of shoulder pain and consider proper diagnostic options such as ultrasonography or MRI.

## CONCLUSIONS

This case implies that muscular atrophy around the scapula and chest wall from chronic quadriparesis may contribute to the development of scapulothoracic bursitis and subsequent periscapular and shoulder pain. In addition, clinician should be alert to it as a possible cause when a patient with quadriparesis complains of shoulder and periscapular pain and consider proper diagnostic options such as ultrasonography or MRI.
